# Energy-saving optimization of the parallel chillers system based on a multi-strategy improved sparrow search algorithm

**DOI:** 10.1016/j.heliyon.2023.e21012

**Published:** 2023-10-17

**Authors:** Xiaodan Shao, Jiabang Yu, Ze Li, Xiaohu Yang, Bengt Sundén

**Affiliations:** a China Northwest Architecture Design and Research Institute, CO. Ltd, Xi'an 710077, Shaanxi Province, China; b Institute of the Building Environment & Sustainability Technology, School of Human Settlements and Civil Engineering, Xi'an Jiaotong University, Xi'an 710049, China; c Lund University, Lund, Sweden

**Keywords:** Chiller load distribution optimization, Multi-strategy improved sparrow search algorithm, Energy consumption saving

## Abstract

The energy usage of parallel chillers systems accounts for 25–40 % of the total energy cost of a building. In light of global warming concerns and the need for energy conservation, it is essential to distribute the load of the parallel chillers systems effectively to achieve energy savings in buildings. Accordingly, this study presents a multi-strategy improved sparrow search algorithm (MSSA) to address optimization of the optimal chillers loading (OCL) problem. The proposed algorithm augments the basic sparrow search algorithm (SSA) by introducing the Sine chaotic map, Levy flight method, and Cauchy variation to enhance diversity, avoid local optima, and increase global and local search capacities. We use 9 benchmark functions to check the performance of the proposed MSSA algorithm, and the results are better than the selected algorithms such as particle swarm algorithm (PSO), harris hawks optimization (HHO), artificial rabbit optimization (ARO) and sparrow search algorithm (SSA). In addition, MSSA is applied to two typical cases to demonstrate its performance to optimal chillers loading and the results indicate that the MSSA outperforms similar algorithms. This study validates that MSSA can provide a promising solution to resolve the OCL problem.

## 1 Introduction

In recent years, energy consumption worldwide has seen a rapid increase [1]. This has contributed significantly to global warming, which is becoming an increasing concern for humanity. If current global warming trends continue, temperatures may rise by 1.5 °C by mid-century. In order to alleviate the great challenges brought by energy consumption to mankind, many scholars devote themselves to the optimization research of energy systems, such as designing more efficient physical models or using artificial intelligence methods such as machine learning to reduce energy cost during operation. Take solar energy heat storage optimization as an example, many scholars use the addition of flip mechanism [2], rotating mechanism [[3], [4], [5]], or the addition of metal foam [6,7], foam-fin structure [8,9], etc., in order to strengthen heat transfer and improve energy utilization efficiency. In addition, many scholars have used machine learning methods to optimize energy systems and have made significant achievements recently. The utilization of machine learning in energy optimization can be broadly divided into two main categories: using neural network to predict energy load and using optimization algorithm to optimize energy system. In terms of load forecasting using neural networks, many scholars have carried out power load forecasting [10], China's future natural gas consumption forecasting [11], office building refrigeration energy consumption forecasting [12], building heat use forecasting [[13], [14], [15]], etc., and trained neural network models to make reasonable forecasts of energy use, so as to formulate appropriate operation strategies to achieve energy-saving goals. In addition, due to the rapid development of meta-heuristic algorithms, many scholars use algorithms to optimize all aspects of energy systems. For example, the optimization of the thermal comfort of the house [16], the optimization of the stability of the nuclear-renewable energy hybrid system [17], the optimization of the utilization of renewable energy in the building [18], the optimization of the equipment capacity configuration of combined heat and power plant [19], the optimization of the energy cost of the HVAC energy terminal [20], the optimization of the energy management of the micro-grid [21], the optimization of the photovoltaic distribution system [22], carbon emission optimization [23], thermo-economic and environmental optimization of solar collector [24], energy subsidy optimization [25], economic transaction optimization of energy system [26], Multi-objective optimization of integrated energy systems [27,28], etc. By using the optimization algorithm to optimize the energy system, the energy system is more economical, energy-saving and stable.

Energy system optimization research has become an important field, and in the field of building refrigeration, due to global warming led to building refrigeration energy consumption continues to rise [29], aim to reduce the energy cost of building refrigeration, the optimization of refrigeration system has become one of the hot spots. Chillers are typical HVAC equipment, widely applied in office buildings [30], commercial buildings [31], and data centers [32], among other public buildings, to provide cold water for air treatment processes. Their energy consumption can account for 25–40 % of the whole energy cost of buildings [33], posing major challenges for energy conservation. The two main energy-saving schemes for chillers are retrofit [34] and operation optimization [35], but retrofitting may necessitate equipment replacement, incurring secondary costs. In contrast, improved operational optimization may achieve more significant energy savings and economic benefits at minimum cost. Parallel chillers systems, composed of chillers with different capacity and performance characteristics [36], could optimize system efficiency and operation flexibility by reasonable distribution of the load, and accordingly achieve energy savings. The optimal chillers loading (OCL) problem, thus, represents a crucial research area for achieving contemporary energy-efficient building operations.

With the rapid development of artificial intelligence recently, a significant number of scholars are committed to optimal chillers loading using various optimization algorithms. As early as the start of this century, some scholars have adopted precise mathematical methods, such as Chinese scholars Chang et al. [37] applied the Lagrange algorithm (LM). However, this method cannot converge when load is low. Aiming to overcome the shortcoming, Chang et al. [38] also utilized the gradient algorithm (GM). While GM exhibits significant convergence when load is low, its search accuracy is inferior. Other precise mathematical methods [39,40] have also been applied to optimal chillers loading. However, achieving high accuracy, convergence, and convergence rates continues to be problematic for precise algorithms in solving OCL problems. To address these shortcomings, many researchers have turned to meta-heuristic algorithms, including GA, which Chang et al. [41,42] employed to optimal chillers loading effectively. Although GA overcomes the limitations of LM, its accuracy must be improved. Accordingly, Chang [43] tested the simulated annealing (SA), which showed more effective performance than GA. Subsequently, other scholars [44,45] proposed the use of the particle swarm optimization (PSO) algorithm to further optimize the load distribution of chillers. Compared to GA and PSO algorithms, the optimization result is significantly superior with more apparent energy-saving advantages in cooling systems having a smaller number of units. The PSO algorithm demonstrates significant advantages in solution accuracy compared to earlier algorithms. However, to further improve algorithmic robustness, Lee et al. [46] applied the differential evolution algorithm (DE) to optimal chillers loading, with results showing that the mean energy consumption value of multiple optimizations is better than PSO. Furthermore, in an effort to improve convergence speed, robustness, and computational complexity, researchers have proposed a range of novel meta-heuristic optimization algorithms, including the improved invasive weed optimization algorithm (EIWO) [47], teaching-learning-based optimization (TLBO) [48], among others. These optimization results demonstrate that meta-heuristic algorithms are effective methods to solve OCL problems. Although many algorithms demonstrate excellent solution accuracy, continued exploration is necessary to identify algorithms with better convergence speed, greater robustness, and computational complexity. This goal is to resolve the OCL problem more accurately, more quickly, and more stably in practical engineering applications.

Sparrow search algorithm (SSA) [49] is an innovative meta-heuristic optimization algorithm founded on group foraging and anti-predation behavior of sparrows by Xue and Shen in 2020. SSA has been highly regarded by numerous researchers [[50], [51], [52]] for its benefits of better search accuracy, quick convergence speed and ease of escaping local optimal solutions. Simultaneously, many scholars have improved SSA to enhance its output further and design more algorithmic versions to solve practical engineering problems. Yuan et al. [53] employed the barycentric reverse learning mechanism in population initialization to improve local search performance, particularly for the optical voltage microgrid system. Ouyang et al. [54] introduced sine and cosine search methods to escape local optimization. Their results demonstrated that the SSA can significantly resolve the problem of support vector machine parameter optimization. Wang et al. [55] integrated the PSO and SSA algorithms to create a hybrid algorithm named PESSA, which performed exceptionally well in UAV path planning problems. These engineering examples validate that, compared to the original algorithm, improved SSA variants offer faster convergence speed with less susceptibility to local optima. Therefore, exploring improvements to initial population or population location update methods to optimal chillers loading, could be beneficial.

To overcome the shortcomings of current algorithms in solving OCL problems, such as low accuracy, slow convergence, high computational complexity, and weak robustness, we proposed a multi-strategy modified sparrow search algorithm (MSSA) to optimize chillers loading. The primary contributions are as follows.

(1) Introduction of the Sine chaotic map, Levy flight method, and Cauchy mutation method to enhance algorithm performance based on the original SSA algorithm.
(2) Nine benchmark functions are used to test the optimization ability of the improved MSSA, and the results illustrate that the improved MSSA has higher search accuracy, faster convergence speed and stronger robustness.
(3) The suitability of MSSA to optimal chillers loading is validated by testing two typical examples, wherein the substantial potential for energy-saving is demonstrated.

The remainder of the paper is arranged as follows: Section 2 describes the physical structure of the parallel chiller system and the mathematical model of the OCL problem. In section 3, the principles of the SSA and MSSA are explained briefly. The testing of benchmark functions for MSSA and the comparison of different metaheuristics are discussed in Section 4. In section 5, two typical Taiwanese cases are analyzed in detail and the superiority of the algorithm over previous methods is demonstrated. Section 6 provides the conclusions and our future expectations.

## 2 Mathematical model

**Fig. 1:**
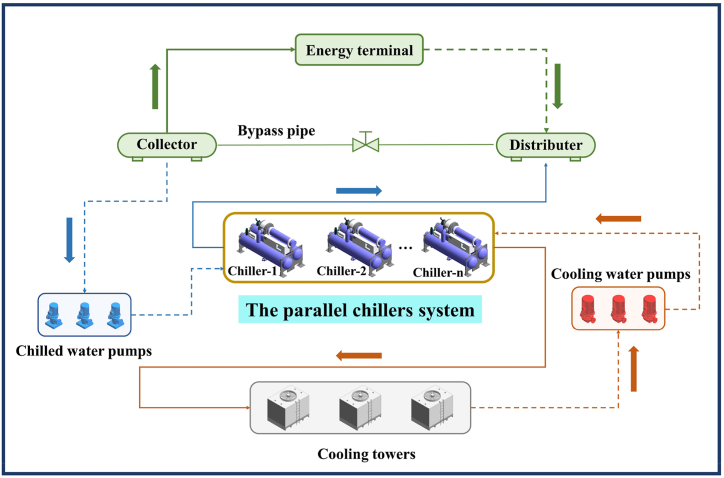
The parallel chillers system.

Fig. 1 presents a common parallel chillers system in a public building, which comprises chillers, cooling towers, pumps and other equipment. The system constitutes multiple chillers of equivalent or different models. After extended operation periods, each chiller unit may exhibit dissimilar performance even if it is of the same type. Since individual cooling units possess varying capacity and energy consumption, we regulate cold-water supply and return water flow control to distribute the system cooling load effectively. If every chiller can each operate at or near optimal efficiency, the entire system will reach optimal efficiency through reasonable load distribution, ultimately achieving energy savings.

Thus, based on the above description, the OCL problems seeks to minimize the entire energy cost of the system through reasonable distribution of the load rate for each chiller unit while ensuring the energy cost demand of terminals. Typically, we can derive the relationship between the part load rate and power cost using the operating data of the chillers, as represented by Equation (1):

(1) $$P_{chiller,i}=d\times PLR_{i}^{3}+c\times PLR_{i}^{2}+b\times PLR_{i}+a$$

where $P_{chiller,i}$ denotes power consumption of the ith chiller unit; *d*, *c*, *b*, and *a* represent the performance parameters of the unit itself; $PLR_{i}$ shows the part load rate, which is the ratio of actual refrigerating capacity to designed refrigerating capacity.

When the *PLR* of the unit is small, it is susceptible to damage, necessitating the constraint of the part load rate. Concurrently, the aggregate load for the system must fulfill the energy demand of the building terminal. The *PLR* constraints and the power equation constraint are represented by Equation (2):

(2) $$\left\{ \begin{array}{c} 0.3\leq PLR\leq 1\;or\;PLR=0\\ \sum_{i=1}^{N}Q_{i}\times PLR_{i}=Q_{demand},i=1,2,3,...,N \end{array}\right.$$

where $Q_{i}$ denotes the designed refrigerating capacity of every chiller; $Q_{demand}$ represents loading demand of building terminals.

Ultimately, the crux of solving the OCL problem lies in determining the minimum energy consumption of the parallel chillers system and the *PLR* of each chiller unit under optimal working conditions. The target equation is expressed as Equation (3):

(3) $$F_{object}=\min\left(\sum_{i=1}^{N}P_{chiller,i}\right)$$

where $F_{object}$ denotes the minimum energy cost of the system.

## 3 Optimization method

### 3.1 Basic sparrow search algorithm (SSA)

The sparrow search algorithm was initially proposed as a meta-heuristic optimization algorithm inspired by sparrows' predation and anti-predation behaviors. It exhibits a robust global search ability and is used extensively to resolve optimization problems across various fields. Sparrows are categorized into producers, scroungers, and scouters.

Initially, *n* sparrows are randomly produced to form the initial sparrow population, with its population matrix equation articulated as Equation (4):

(4) $$X=\left[\begin{array}{cccc} x_{1,1} & x_{1,2} & \cdots & x_{1,m}\\ x_{2,1} & x_{2,2} & \cdots & x_{2,m}\\ \vdots & \vdots & \vdots & \vdots \\ x_{n,1} & x_{n,2} & \cdots & x_{n,m} \end{array}\right]$$

where *n* denotes the quantity of sparrows and *m* is decision variable dimension of the problem that needs to be solved.

The initial fitness is calculated as Equation (5):

(5) $$F_{X}=\left[\begin{array}{c} f\left(x_{1,1}\;x_{1,2}\cdots x_{1,m}\right)\\ f\left(x_{2,1}\;x_{2,2}\cdots x_{2,m}\right)\\ \vdots \vdots \cdots \vdots \\ f\left(x_{n,1}\;x_{n,2}\cdots x_{n,m}\right) \end{array}\right]$$

Producers exhibit superior fitness values, granting them priority access to food. They are responsible for foraging throughout the sparrow population, providing foraging direction. The position updates are presented in Equation (6):

(6) $$X_{i,j}^{t+1}=\left\{ \begin{array}{c} X_{i,j}^{t}\cdot \exp\left(-\frac{i}{\alpha \cdot iter_{\max}}\right)\;when\;R_{2}<\;ST\\ X_{i,j}^{t}+Q\cdot L\;when\;R_{2}\;>\;ST \end{array}\right.$$

where $X_{i,j}^{t}$ illustrates the position of the previous generation of individual; α denotes a random number within the range of (0,1]; $R_{2}\in \left[0,1\right]$ and ST∈[0.5,1] denote a warning value and a safety value, separately; Q represents a set of random number that follows a normal distribution and L is a matrix of 1×m.

Scroungers in sparrows’ population continuously monitor producer activities. If producers discover better food, scroungers compete for it, receiving food upon success. In contrast, if they fail, they fly to other territories in search of nourishment. Scrounger position updates are expressed by Equation (7):

(7) $$X_{i,j}^{t+1}=\left\{ \begin{array}{c} Q\cdot \exp\left(\frac{X_{worst}-X_{i,j}^{t}}{i^{2}}\right)\;when\;i\;>\;n/2\\ X_{p}^{t+1}+\left|X_{i,j}^{t}-X_{p}^{t+1}\right|\cdot A^{+}\cdot L\;otherwise \end{array}\right.$$

where $X_{worst}$ denotes the worst position and $X_{p}^{t+1}$ represents the position of the best individuals in the next generation; $A^{+}=A^{T}{\left(AA^{T}\right)}^{-1}$, with A denoting a 1×m matrix whose elements are 1 or -1.

When a scouter detects them to be at risk, it departs the current area and flies to the best area. Their position updates are shown in Equation (8):

(8) $$X_{i,j}^{t+1}=\left\{ \begin{array}{c} X_{best}^{t}+\beta \cdot \left|X_{i,j}^{t}-X_{best}^{t}\right|\;when\;f_{i}\;>\;f_{g}\\ X_{i,j}^{t}+K\left(\frac{\left|X_{i,j}^{t}-X_{worst}^{t}\right|}{\left(f_{i}-f_{w}\right)+\varepsilon }\right)\;when\;f_{i}=f_{g} \end{array}\right.$$

where $X_{best}^{t}$ is the current global best position; β is the step size control value that is a normally distributed random number whose the mean is zero and the variance is 1; K∈[−1,1] denotes a random number; $f_{i}$, $f_{g}$ and $f_{w}$ are the fitness value of current individual, the optimal value, and the current worst value, respectively; ε denotes an extremely small constant.

### 3.2 Multi-strategy improved sparrow search algorithm (MSSA)

Notwithstanding the potential of SSA to tackle the OCL problem, there is still considerable scope for enhancing search accuracy and convergence speed. Hence, we propose a multi-strategy improved sparrow search algorithm (MSSA), which introduces the Sine chaotic map for population initialization, Levy flight mechanism to improve producer location, and Cauchy mutation to enhance the scroungers' search capabilities in SSA. The details are outlined in this section.

#### 3.2.1 Sine chaotic method of population initialization

**Fig. 2:**
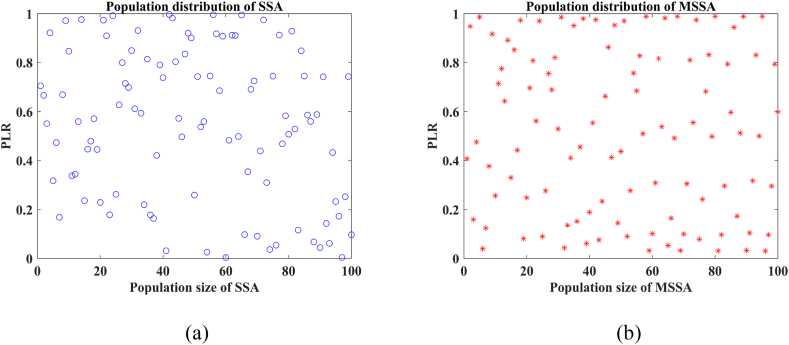
Population distribution of SSA and MSSA. (a) SSA. (b) MSSA.

Fig. 2(a) illustrates the individual distribution mode of the randomly-generated population by the basic SSA algorithm. Random particle distributions in the solution space may lead to the failure to identify optimal values within a small search range or slow convergence.

To amplify the population diversity, the Sine chaotic map is introduced to produce the sparrow population and its population generation mode is expressed by Equation (9):

(9) $$X_{i,j}^{t+1}=a\cdot \sin\left(\pi X_{i,j}^{t}\right),a\in \left[0,1\right]$$

Fig. 2(b) displays the population distribution after integrating the Sine chaotic map. Compared to Fig. 2(a), particle distribution within the solution space is more uniform, improving the comprehensiveness of understanding and augmenting global search efficacy during the initial iteration stages.

#### 3.2.2 Improvement of producers’ position

The Levy flight strategy mimics birds' flight trajectory, characterized by random alternating between long and short distances. As the primary optimization group, producers leverage the Levy flight method to enhance the global and local search capabilities of the algorithm, facilitating quick convergence to the optimal value. The updated producer location is expressed in Equation (10):

(10) $$X_{i,j}^{t+1}=\left\{ \begin{array}{c} X_{i,j}^{t}\cdot \exp\left(-\frac{i}{\alpha \cdot iter_{\max}}\right)\cdot levy\left(m\right)\;when\;R_{2}<\;ST\\ X_{i,j}^{t}+L\cdot levy\left(m\right)\;when\;R_{2}\;>\;ST \end{array}\right.$$

where the Levy flight mechanism is represented in Equation (11):

(11) $$levy\left(m\right)=0.01\times \frac{r_{1}\times \sigma }{{\left|r_{2}\right|}^{\left(1/\xi \right)}}$$

where $r_{1},r_{2}\in \left[0,1\right]$ are two random numbers; the value of ξ is usually 1.5; σ is represented as shown in Equation (12):

(12) $$\sigma ={\left(\frac{\Gamma \left(1+\xi \right)\times \sin\left(\pi \xi /2\right)}{\Gamma \left(\left(1+\xi \right)/2\right)\times \xi \times 2^{\left(\left(\xi -1\right)/2\right)}}\right)}^{\left(1/\xi \right)}$$

#### 3.2.3 Improvement of scroungers’ position

Scroungers serve as the secondary foragers in the sparrow population, with their position update mode impacted by producers' current optimal value. To prevent scroungers from getting trapped in a local optimal value, the Cauchy variation is introduced to perturb their position updating, taking advantage of the characteristics of the Cauchy distribution that it has a small peak value at the origin and long distribution at both ends. Our goal is to improve the local search ability and the quality of the optimal solution [56]. If the search ability of the algorithm is strengthened, then every optimization can find a very competitive solution, which indirectly enhances the robustness of the algorithm, making the difference between the results of each optimization of the algorithm very small. The enhanced location update method of scroungers is represented below in Equation (13):

(13) $$X_{i,j}^{t+1}=\left\{ \begin{array}{c} Q\cdot \exp\left(\frac{X_{worst}-X_{i,j}^{t}}{i^{2}}\right)\;when\;i\;>\;n/2\\ X_{p}^{t+1}\cdot \left(1+cauchy\left(0,1\right)\right)+\left|X_{i,j}^{t}-X_{p}^{t+1}\cdot \left(1+cauchy\left(0,1\right)\right)\right|\cdot A^{+}\cdot L\;otherwise \end{array}\right.$$

where cauchy(0,1) is Cauchy variation.

#### 3.2.4 The implementation of MSSA

**Fig. 3:**
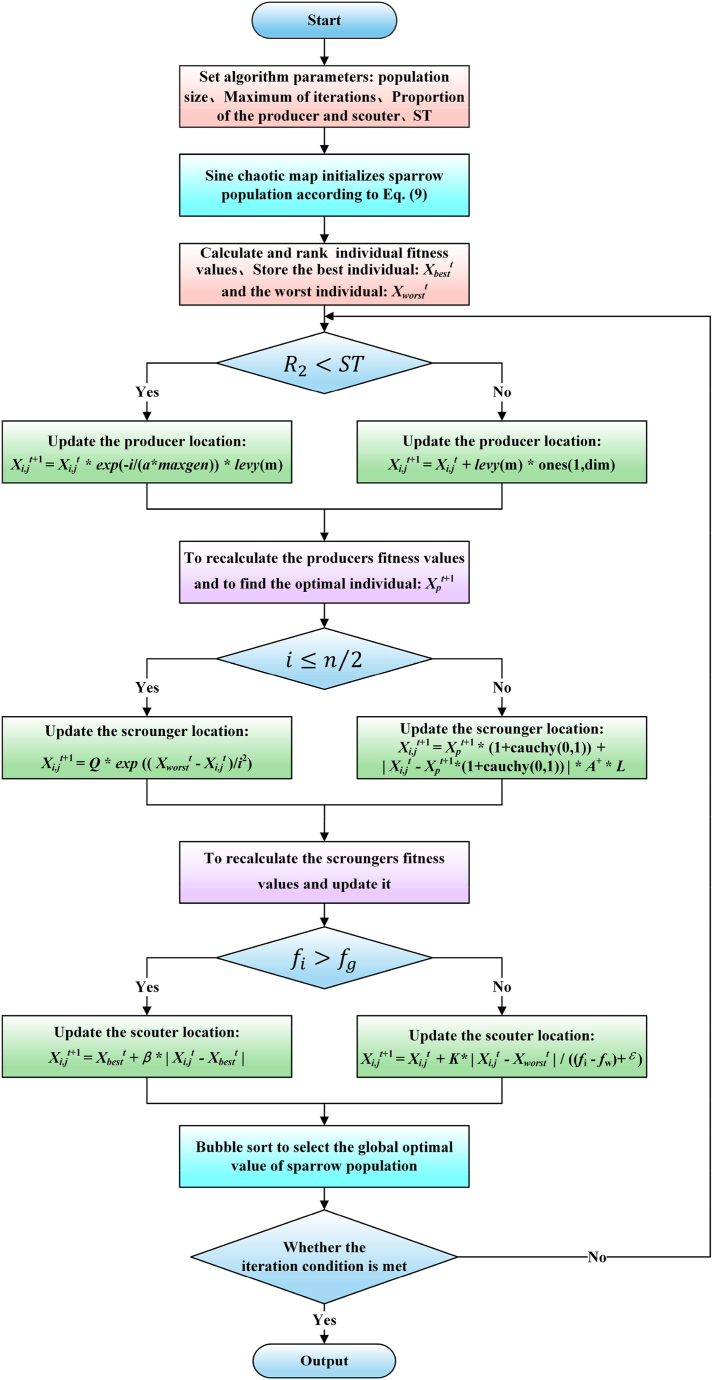
Calculation flow of MSSA.

Fig. 3 illustrates the flow chart of MSSA, as detailed below.

(1) Basic algorithm parameters such as the size of population, the maximum number of iterations is initialized.
(2) The Sine chaotic map generates the initial population.
(3) The fitness value of sparrows is calculated and sorted.
(4) When $R_{2}\in \left[0,1\right]$, the Levy flight method is adopted to improve producers' positions.
(5) When $f_{i}\;>\;f_{g}$, the Cauchy variation perturbs scroungers' positions.
(6) Scouters' positions are updated according to Equation (8).
(7) The optimal fitness value and position of the current population are updated.
(8) Determine if the termination criterion is satisfied. When satisfied, output the result.

### 3.3 The framework to optimal chillers loading by MSSA

**Fig. 4:**
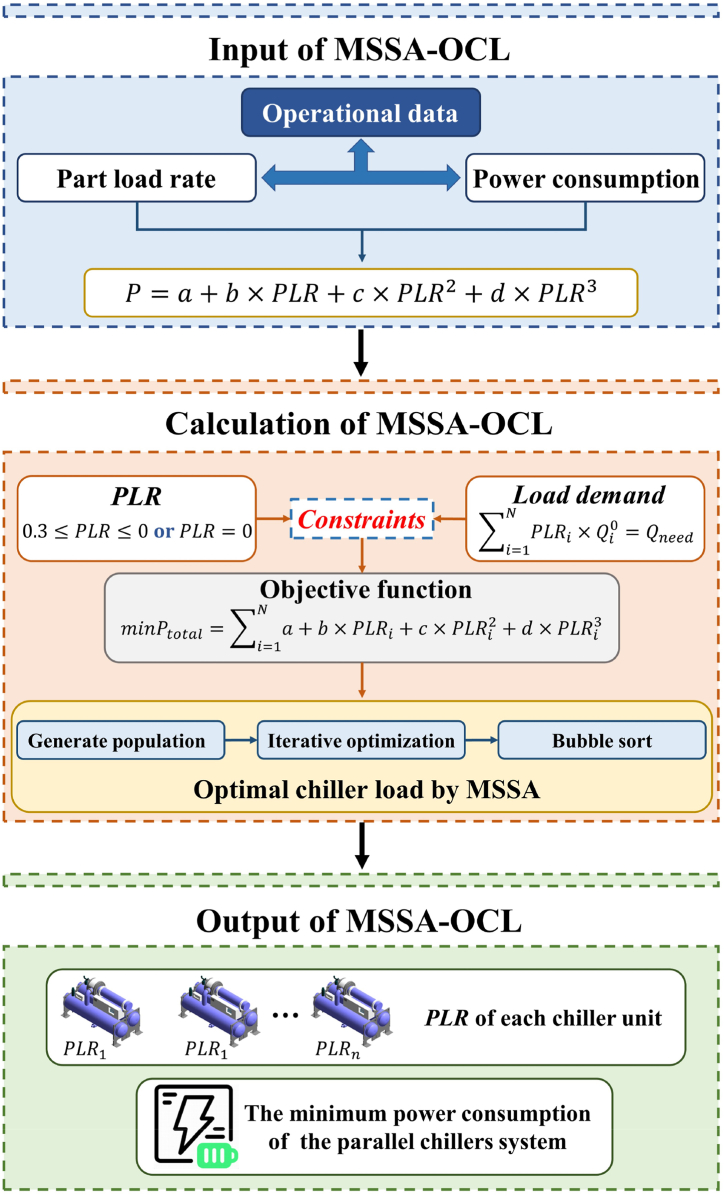
The framework to optimal chillers loading by MSSA.

To enhance our understanding of how to use the MSSA algorithm to tackle the OCL problem, Fig. 4 depicts its framework, which can be divided into three parts. Firstly, we accurately fit the load rate of the chiller polynomial using historical operation data, such as cooling capacity and energy consumption recorded at a specific time. Next, we formulate the constraints for the chiller load rate and building terminal energy mathematically, followed by a detailed specification of relevant algorithm parameters and iterative solutions. Finally, we employ MSSA to output the energy cost and *PLR* of each chiller.

## 4 Experimental results and discussion

To verify the performance of the proposed MSSA, we tested it applying nine well-known benchmark functions and compared the results with the PSO, HHO, ARO, and SSA algorithms. First, we give the details of benchmark function and the setting of the test algorithms, and then we analyze the search accuracy, convergence speed, robustness in detail. In order to ensure a consistent testing environment, Windows 11 is utilized as the operating system, with an Intel Core (TM) i7-4790K@4.0 processor and 64 GB of memory and MATLAB R2021a is testing tool.

### 4.1 Details of benchmark functions and parameter settings

**Fig. 5:**
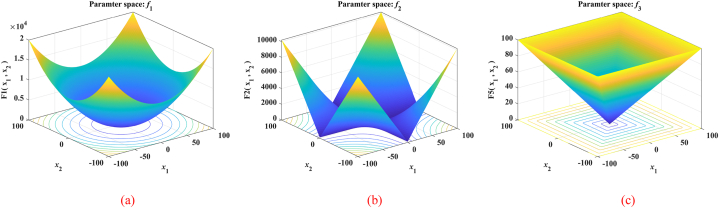
3D data diagram of unimodal functions. (a) *f*_1_. (b) *f*_2_. (c) *f*_3_.

**Fig. 6:**
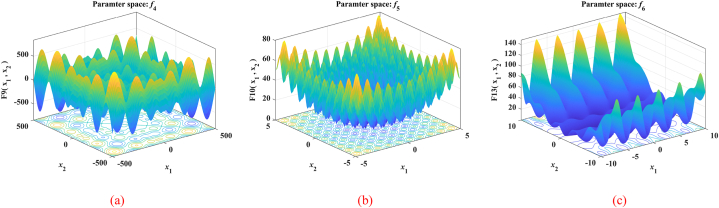
3D data diagram of multimodal functions. (a) *f*_4_. (b) *f*_5_. (c) *f*_6_.

**Fig. 7:**
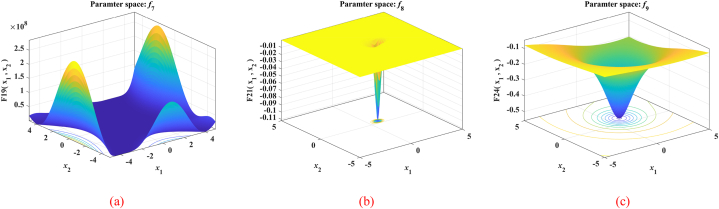
3D data diagram of fixed-dimension functions. (a) *f*_7_. (b) *f*_8_. (c) *f*_9_.

The nine benchmark functions used in the tests are unimodal functions (see Fig. 5(a), (b) and (c)), multimodal functions (see Fig. 6(a), (b) and (c)), and fixed-dimensional functions (see Fig. 7(a), (b) and (c)). Unimodal function exists only one optimal solution globally, which is applied to verify the convergence and development capability of the algorithm. Multimodal function easily causes the algorithm to be in local optimal because of the existence of many local optimal solutions, so the use of multimodal functions is to verify the global and local search capability. What's more, in order to test the performance of the proposed MSSA more comprehensively, we also use three representative fixed-dimension functions. The detail information of 9 benchmark functions is illustrated in Table 1, Table 2 and Table 3 respectively.

**Table 1:** Details for unimodal functions.

Name	Function	D	Range	$f_{best}$
Sphere	$F_{1}\left(x\right)=\sum_{i=1}^{D}x_{i}^{2}$	30	[-100,100]	0
Schwefel's 2.22	$F_{2}\left(x\right)=\sum_{i=1}^{D}\left|x_{i}\right|+\prod_{i=1}^{D}\left|x_{i}\right|$	30	[-10,10]	0
Schwefel's 2.21	$F_{3}\left(x\right)=\max_{i}\left\{\left|x_{i}\right|,1\leq i\leq D\right\}$	30	[-100,100]	0

**Table 2:** Details for Multimodal functions.

Name	Function	D	Range	$f_{best}$
Schwefel	$F_{4}\left(x\right)=\sum_{i=1}^{D}\left(-x_{i}\cdot \sin\sqrt{\left|x_{i}\right|}\right)$	30	[-500,500]	−12569.5
Rastrigin	$F_{5}\left(x\right)=\sum_{i=1}^{D}\left[x_{i}^{2}-10\;\cos\left(2\pi x_{i}\right)+10\right]$	30	[-5.12,5.12]	0
Penalized	$\begin{array}{l} F_{6}\left(x\right)=\frac{\pi }{D}\{10\;\sin\left(\pi x_{1}\right)+\sum_{i=1}^{D-1}{\left(x_{i}-1\right)}^{2}\left[1+10\;\sin^{2}\left(\pi x_{i+1}\right)\right]\\ +{\left(x_{D}-1\right)}^{2}\}+\sum_{i=1}^{D}u\left(x_{i},10,100,4\right) \end{array}$	30	[-50,50]	0

**Table 3:** Details for Fixed-dimension functions.

Name	Function	D	Range	$f_{best}$
Goldstein-Price	$\begin{array}{l} F_{7}\left(x\right)=[1+{\left(x_{1}+x_{2}+1\right)}^{2}\times (19-14x_{1}+3{x_{1}}^{2}\\ -14x_{2}+6x_{1}x_{2}+3{x_{2}}^{2})]\times [30+{\left(2x_{1}-3x_{2}\right)}^{2}\times \\ \left(18-32x_{1}+12{x_{1}}^{2}+48x_{2}-36x_{1}x_{2}+27{x_{2}}^{2}\right)] \end{array}$	2	[-2,2]	3
Hartman's 6	$F_{8}\left(x\right)=-\sum_{i=1}^{4}c_{i}\;\exp\left(-\sum_{j=1}^{6}a_{ij}{\left(x_{j}-p_{ij}\right)}^{2}\right)$	6	[0,1]	−3.322
Shekel's 10	$F_{9}\left(x\right)=-\sum_{i=1}^{10}{\left[\left(X-a_{i}\right){\left(X-a_{i}\right)}^{T}+c_{i}\right]}^{-1}$	4	[0,10]	−10.5364

In addition, we also select some commonly used algorithms such as PSO [57], HHO [58], ARO [59] and SSA [49] to compare the capability of the proposed MSSA algorithm. Aim to make the experiment more reliable, each algorithm carried out 30 independent operations on each benchmark function, the size of population is set to 30, and the maximum iterations is 200. Table 4 describes the parameter details of the 5 algorithms. After testing, we get the best value, mean value, maximum value and standard deviation represented in Table 5. The mean value denotes the search capability of the test algorithm while the standard deviation means the robustness.

**Table 4:** Algorithm parameter setting.

Algorithm	Parameters	Values
PSO	Cognitive and social constant,	$c_{1}=c_{2}=2$
HHO	Possibility thresholds of escaping, escaping energy	0.5, 0.5
ARO	No special parameters	–
SSA	Ratio of producers and scouters, the safety threshold	PD=20%,SD=20%,ST=0.8
MSSA	Ratio of producers and scouters, the safety threshold	PD=20%,SD=20%,ST=0.8

**Table 5:** Results of 9 benchmark functions of 5 algorithms.

Fun	Value	PSO	HHO	ARO	SSA	MSSA
F1	Best	77.591	6.014 × 10^−52^	1.557 × 10^−26^	9.803 × 10^−292^	0
	Mean	163.857	2.822 × 10^−41^	1.820 × 10^−19^	6.142 × 10^−59^	0
	Worst	304.911	8.456 × 10^−40^	4.565 × 10^−18^	1.784 × 10^−57^	0
	SD	51.392	1.518 × 10^−40^	8.193 × 10^−19^	3.201 × 10^−58^	0
F2	Best	7.606	5.717 × 10^−27^	8.860 × 10^−15^	0	0
	Mean	39.790	6.562 × 10^−23^	2.239 × 10^−12^	3.759 × 10^−26^	0
	Worst	82.114	1.526 × 10^−21^	1.258 × 10^−11^	1.128 × 10^−24^	0
	SD	16.940	2.743 × 10^−22^	3.538 × 10^−12^	2.024 × 10^−25^	0
F3	Best	7.464	1.495 × 10^−27^	1.425 × 10^−11^	4.513 × 10^−286^	0
	Mean	12.884	2.700 × 10^−21^	2.718 × 10^−8^	7.270 × 10^−29^	0
	Worst	20.689	4.551 × 10^−20^	2.650 × 10^−7^	2.137 × 10^−27^	0
	SD	3.069	9.837 × 10^−21^	6.348 × 10^−8^	3.835 × 10^−28^	0
F4	Best	−7869.4	−12569.5	−9924.6	−8777.9	−12569.5
	Mean	−6224.4	−12439.7	−8949.5	−7456.1	−12484.8
	Worst	−4592.6	−9007.8	−8127.3	−5958.8	−11370.1
	SD	856.9	638.6	459.1	610.4	219.2
F5	Best	208.774	0	0	0	0
	Mean	260.980	0	0	0	0
	Worst	318.282	0	0	0	0
	SD	24.914	0	0	0	0
F6	Best	7.933	7.272 × 10^−8^	0.001	1.511 × 10^−10^	2.416 × 10^−10^
	Mean	20.057	4.372 × 10^−5^	0.008	4.475 × 10^−8^	4.799 × 10^−8^
	Worst	58.565	2.722 × 10^−4^	0.017	4.488 × 10^−7^	3.657 × 10^−7^
	SD	10.487	6.881 × 10^−5^	0.005	8.526 × 10^−8^	7.073 × 10^−8^
F7	Best	3.0001	3.0000	3.0000	3.0000	3.0000
	Mean	3.0028	3.0000	3.0000	7.5000	3.0000
	Worst	3.0247	3.0003	3.0000	30.0000	3.0000
	SD	0.0049	5.19 × 10^−5^	0	10.0623	0
F8	Best	−3.322	−3.252	−3.322	−3.322	−3.322
	Mean	−3.055	−3.036	−3.282	−3.278	−3.310
	Worst	−1.706	−2.639	−3.203	−3.203	−3.203
	SD	0.300	0.159	0.056	0.057	0.036
F9	Best	−10.4883	−10.4261	−10.5364	−10.5364	−10.5364
	Mean	−9.0932	−5.3507	−10.1732	−8.9097	−10.5364
	Worst	−2.4170	−2.4007	−5.1193	−5.1285	−10.5364
	SD	2.1574	1.3715	1.3501	2.4755	2.91 × 10^−6^

### 4.2 Discussion of search accuracy and robustness

Table 5 represents the benchmark function test results of 5 algorithms. For the unimodal function F1–F3, there is and only MSSA can search for the optimal value in 30 tests, showing very strong development ability. MSSA algorithm can get the optimal value in every test. What's more, the results of MSSA and SSA are competitive compared with other algorithms, but the standard deviation of MSSA is smaller than SSA, which proves the Cauchy variation strategy effectively enhances the robustness of the algorithm.

In the test of F4–F6 in multimodal functions, MSSA also describe significantly excellent performance. Due to the existence of many local optimal for multimodal functions, the algorithm is easily stuck in the optimization solution, so whether the algorithm can avoid being in the local optimal solution and search the global best value is an important index to measure its exploration performance. Especially for F4, only MSSA and HHO algorithms can find the optimal solution, but the mean value of MSSA is smaller than HHO. In addition, SSA algorithm did not search the best value in the F4 test, while MSSA introduced Levy strategy in the producer's position update, which enhanced the local search capability of MSSA. Therefore, compared with SSA, MSSA can effectively avoid local optimal and search the global optimal solution.

Finally, the test results of F7–F9 in fixed-dimension functions further describe that MSSA has excellent searching ability and robustness. The mean value and standard deviation of MSSA are significantly better than those of the four algorithms, which also proves that our improved strategy is significantly effective to improve the robustness and the search accuracy.

### 4.3 Discussion of convergence speed

**Fig. 8:**
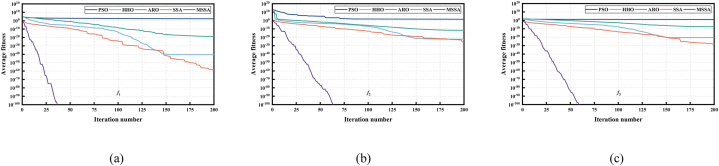
Convergence curve of 5 algorithms in unimodal functions tests. (a) *f*_1_. (b) *f*_2_. (c) *f*_3_.

**Fig. 9:**
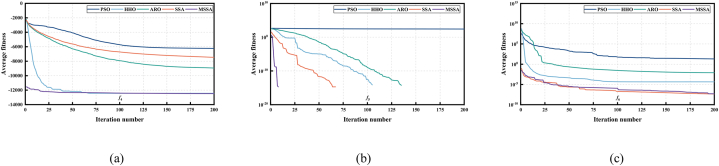
Convergence curve of 5 algorithms in multimodal functions tests. (a) *f*_4_. (b) *f*_5_. (c) *f*_6_.

**Fig. 10:**
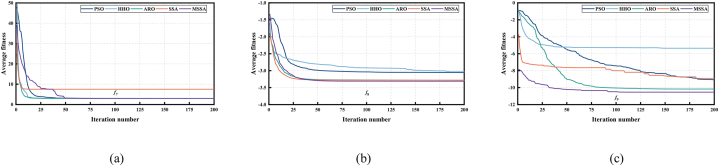
Convergence curve of 5 algorithms in fixed-dimension functions tests. (a) *f*_7_. (b) *f*_8_. (c) *f*_9_.

Fig. 8, Fig. 9, Fig. 10 shows the convergence capability of 9 benchmark function tests. As shown in Fig. 8(a), (b) and (c), for F1–F3 of unimodal functions, we can intuitively find that the convergence rate of MSSA is extremely fast. Compared with SSA and other algorithms, MSSA can converge to a competitive optimal value in the early or middle search stage. The reason is that MSSA uses sine chaotic mapping to enrich the diversity of the population, so that the algorithm has a greater possibility to obtain the best solution in the early iteration, thus speeding up the entire convergence process.

Fig. 9 illustrates the convergence capability in multimodal function tests. For F4, only MSSA and HHO can converge to a competitive solution in the whole iteration process, but MSSA converges faster than HHO algorithm in Fig. 9(a). In addition, except for the PSO algorithm, other algorithms can converge to the optimal value "0″ in F5 test, however, Fig. 9(b) demonstrates MSSA uses the least number of iterations, and the convergence is completed in about 10 generations. For F6, Fig. 9(c) presents that only SSA and MSSA can converge to a competitive superior value. The above results show that MSSA can be well applied to the solution of multimodal functions, and a competitive solution can be obtained.

Finally, Fig. 10 illustrates the convergence curve for the fixed-dimension function test. For F7 and F8, Fig. 10(a) and (b) illustrate that MSSA algorithm can converge to a better solution in the early search stage, thus speeding up the global convergence speed. In the F9 test, in the early search stage, only MSSA quickly converges to the optimal value in Fig. 10(c), while other comparison algorithms fail to search for the optimal value, which fully illustrates that MSSA has excellent convergence speed and solving accuracy.

### 4.4 Discussion of computational complexity

In order to further verify the improved effect of the algorithm, we select the computational time used by the algorithm to solve the benchmark function to demonstrate the computational complexity of the algorithm. The shorter the time, the lower the computational complexity of the algorithm and the better the computational performance. Table 6 shows the cumulative running time of 30 operations for 9 benchmark functions tested by 5 algorithms. For F1, F2, F4, F6, F7 and F8, MSSA takes slightly longer time than PSO, but the search accuracy and convergence speed of MSSA are more competitive than PSO, in general, MSSA is better than PSO. For F3, F5 and F9, MSSA requires the least computation time. In addition, the calculation time of MSSA is also reduced compared with SSA, which means that the introduction of improvement strategies effectively improves the calculation performance of MSSA and makes MSSA have a faster calculation speed.

**Table 6:** Cumulative running time of 5 algorithms in 9 benchmark functions tests.

	Cumulative running time (s)				
	PSO	ARO	HHO	SSA	MSSA
F1	1.776	2.424	2.324	1.854	1.789
F2	1.748	2.355	2.135	1.891	1.883
F3	1.797	2.329	2.217	1.866	1.789
F4	1.853	2.473	2.707	2.012	1.960
F5	1.891	2.336	2.494	1.943	1.866
F6	3.533	4.168	6.545	3.983	3.927
F7	1.590	2.090	2.084	1.679	1.655
F8	1.722	2.220	2.326	1.883	1.802
F9	2.111	2.395	2.774	2.100	2.053

## 5 Application of MSSA

### 5.1 Test cases

In order to test the efficacy of the MSSA to optimal chillers loading, we selected two typical cases in this study. After an extended period of operation, the performance of each chiller within these two systems differs significantly. Table 7 provides the detail coefficients of each chiller in the two systems.

**Table 7:** The details of two parallel chillers system.

System	Unit code	a	b	c	d	Capacity/RT
	1	399.345	−122.12	770.46	–	1280
	2	287.116	80.04	700.48	–	1280
Case 1	3	−120.505	1525.99	−502.14	–	1280
	4	−19.121	898.76	−98.15	–	1280
	5	−95.029	1202.39	−352.16	–	1250
	6	191.750	224.86	524.04	–	1250
	1	104.09	166.57	−430.13	512.543	450
Case 2	2	−67.15	1177.79	−2174.53	1456.53	450
	3	384.71	−779.13	1151.42	−63.20	1000
	4	541.63	413.48	−3626.50	4021.41	1000

To ensure a consistent testing environment, Windows 11 is utilized as the operating system, with an Intel Core (TM) i7-4790K@4.0 processor and 64 GB of memory. We employ MATLAB R2021a as our testing tool. The population size of all tested algorithms is 100, the number of iterations is 100, and the settings of other parameters are shown in Table 4.

### 5.2 Searching accuracy analysis

In the two cases, the results of MSSA were compared with GA [42], PSO [45], SSA [60], ARO [59] and HHO [58], which are represented in Table 8 and Table 9, severally.

**Table 8:** The minimum energy cost of GA, PSO, SSA, ARO, HHO and MSSA of Case 1.

Load	GA [42]	PSO [45]	SSA [60]	ARO	HHO	MSSA	Energy saving (kW)				
Demand	P/kW(A)	P/kW(B)	P/kW(C)	P/kW(D)	P/kW(E)	P/kW(F)	(F-A)	(F–B)	(F–C)	(F-D)	(F-E)
6858RT	4766.33	4739.78	4768.23	4739.91	4738.58	4738.58	−27.75	−1.20	−29.65	−1.34	0.00
6477RT	4459.16	4423.05	4443.71	4422.30	4421.71	4421.65	−37.51	−1.40	−22.06	−0.66	−0.06
6096RT	4185.87	4147.81	4160.34	4143.89	4143.71	4143.71	−42.16	−4.10	−16.63	−0.18	0.00
5717RT	3940.60	3920.96	3896.26	3843.02	3842.55	3842.55	−98.05	−78.41	−53.71	−0.47	0.00
5334RT	3706.22	3642.58	3625.57	3546.65	3546.44	3546.44	−159.78	−96.14	−79.13	−0.21	0.00

**Table 9:** The minimum energy cost of GA, PSO, SSA, ARO, HHO and MSSA of Case 2.

Load	GA [42]	PSO [45]	SSA [60]	ARO	HHO	MSSA	Energy saving (kW)				
Demand	P/kW(A)	P/kW(B)	P/kW(C)	P/kW(D)	P/kW(E)	P/kW(F)	(F-A)	(F–B)	(F–C)	(F-D)	(F-E)
2610RT	1862.18	1857.30	1868.26	1857.30	1857.30	1857.30	−4.88	0.00	−10.96	0.00	0.00
2320RT	1457.23	1455.66	1469.05	1455.66	1455.67	1455.66	−1.57	0.00	−13.39	0.00	−0.01
2030RT	1183.80	1178.14	1187.24	1178.14	1178.14	1178.14	−5.66	0.00	−9.10	0.00	0.00
1740RT	1001.62	998.53	1081.61	998.53	998.54	998.53	−3.09	0.00	−83.08	0.00	0.00
1450RT	907.72	820.07	852.53	820.07	820.07	820.07	−87.65	0.00	−32.46	0.00	0.00
1160RT	856.30	651.07	686.65	651.07	651.07	651.07	−205.23	0.00	−35.58	0.00	0.00

As illustrated in Table 8, MSSA displayed excellent search accuracy performance when applied to Case 1 with multiple chillers. MSSA achieved an energy-saving potential of about 27.75 kW–159.78 kW compared to GA algorithm. It can be found that MSSA saves energy consumption by 1.21 kW–96.14 kW compared with the test results of PSO, with the maximum relative energy saving rate of 2.64 % when load is 5334RT. In addition, compared with the unimproved SSA, MSSA can save 16.63 kW–79.13 kW of energy consumption. What's more, compared with ARO and HHO algorithms, MSSA has no obvious energy-saving advantages, thus we believe that the search accuracy of these three algorithms is comparable when solving OCL problems.

Table 9 reveals that in all working conditions with fewer chillers, the optimization results of MSSA algorithm are superior to GA and SSA algorithm. Compared with the test results of GA, MSSA can achieve a maximum energy saving of 205.23 kW at a load of 5334RT. And compared with SSA, the improved MSSA can save 9.10 kW–83.08 kW of energy consumption, and its relative energy saving rate is 0.59 %–7.68 %. However, compared to PSO, ARO and HHO, MSSA did not produce any energy savings. By analyzing the results of case 1 and case 2, we can conclude.

(1) MSSA algorithm is better suited for systems with a large number of chillers. Compared to commonly used optimization algorithms such as GA, PSO and SSA, MSSA consistently demonstrates a distinct energy-saving potential.
(2) When the building energy consumption demand is at a low level, the energy saving potential of MSSA is greater.

Similar to the characteristics of the previous benchmark function test performance, compared with SSA, because MSSA introduces levy flight method to enhances the search capability, MSSA can obtain competitive solutions with higher accuracy in OCL problems compared with SSA. In addition, as many decision variables and complex constraint conditions of OCL problem, it is very difficult for us to obtain the theoretical reference best value by mathematical method. However, the test results of two cases show that the calculation results of MSSA algorithm are equal to or better than many existing algorithms, so we believe that MSSA can provide a relatively optimal solution for OCL problem at present. In a word, as HVAC systems of most public buildings function at low load levels for most of the time, MSSA algorithm has significant energy-saving potential in HVAC systems operation.

### 5.3 Convergence analysis

For the evaluation of OCL algorithm performance, we believe that the search accuracy is the most important, followed by the convergence speed. If an algorithm cannot solve a competitive solution, it means that it is not necessary to compare its convergence speed with other algorithms with higher search accuracy.

Compared to the GA, PSO and SSA algorithms, the MSSA algorithm displays unparalleled search accuracy. To further validate the advantages of the MSSA algorithm in tackling OCL problems, we compare it with the ARO [59] and HHO [58] algorithms, which possess similar excellent solving accuracy to the MSSA algorithm, and analyze and compare them from the aspects of convergence speed, computational complexity, and robustness. To ensure a fair test, the population number and iteration times of each algorithm were identically set and evaluated independently 30 times under the same computational environment. (The population size is 100, the number of iterations is 100, and other parameters are shown in Table 4)

**Fig. 11:**
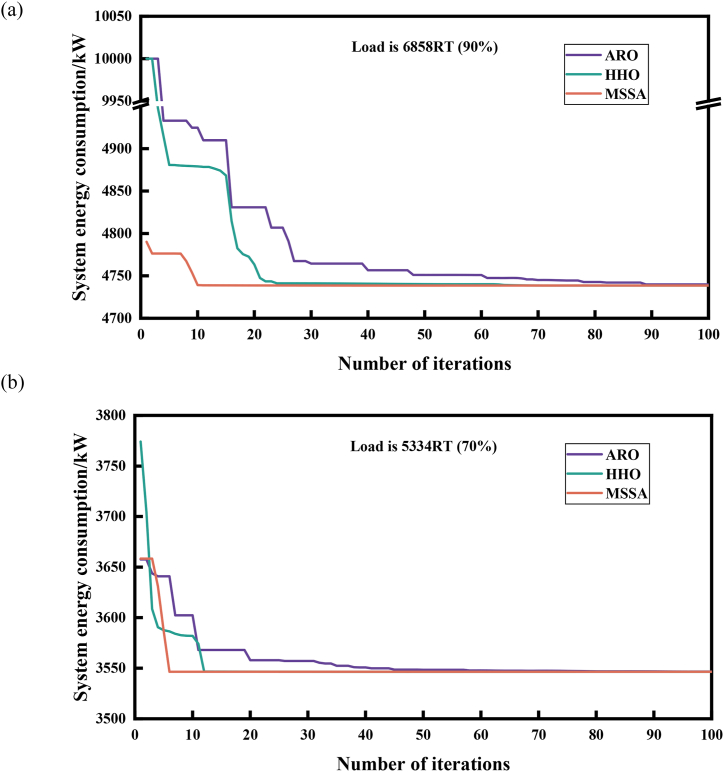
Comparison of convergence speed of 3 algorithms of Case 1. (a) Load is 6858RT. (b) Load is 5334RT

**Fig. 12:**
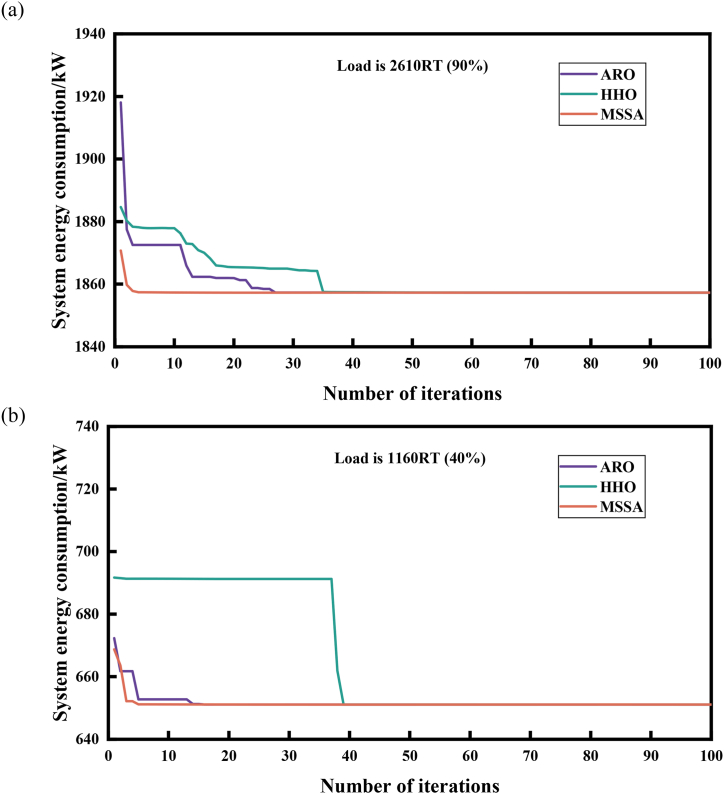
Comparison of convergence speed of 3 algorithms of Case 2 (a) Load is 2610RT. (b) Load is 1160RT

To obtain a comprehensive understanding of the convergence capability of the MSSA, we selected one high-load demand condition and one low-load demand condition for each case. Fig. 11 displays the convergence curve of the three algorithms for the load demand of 6858RT and 5334RT in Case 1. In Fig. 11(a), when the load is 6858RT, the three algorithms converge towards the optimal value within 100 iterations. However, ARO algorithm takes 90 generations to achieve convergence, while HHO algorithm takes around 70 generations. In contrast, the MSSA algorithm completes its convergence within 10 generations, resulting in an 88.9 % reduction in the number of iterations compared to ARO and an 85.7 % reduction compared to HHO. Under the low-load demand condition of 5334RT, ARO algorithm requires around 80 generations to reach the optimal value, HHO algorithm takes roughly 10–15 generations, while MSSA algorithm converges within 10 generations, displaying an exceptionally rapid convergence rate in Fig. 11(b). In Case 2, which contains a small number of coolers in the system, at 2610RT with a high load demand, Fig. 12(a) depicts that it requires about 30 generations for ARO to converge to the optimal value, and 35 generations for HHO, whereas MSSA completes the convergence within 10 generations, resulting in a 66.7 % reduction compared to ARO and a 71.4 % reduction compared to HHO. Moreover, when the load is 1160RT, MSSA can converge to the optimal value within five generations compared to ARO and HHO algorithms, as illustrated in Fig. 12(b). Since algorithms compared with MSSA adopts the random generation method during the initial population generation, while MSSA utilizes the Sine chaotic mapping strategy, in contrast, this strategy enriches the distribution range of particles in the solution space, making MSSA have a significant advantage to approach the optimal solution in the early stage of iteration, so as to rapidly converge to a highly competitive stable value.

### 5.4 Computational complexity analysis

**Fig. 13:**
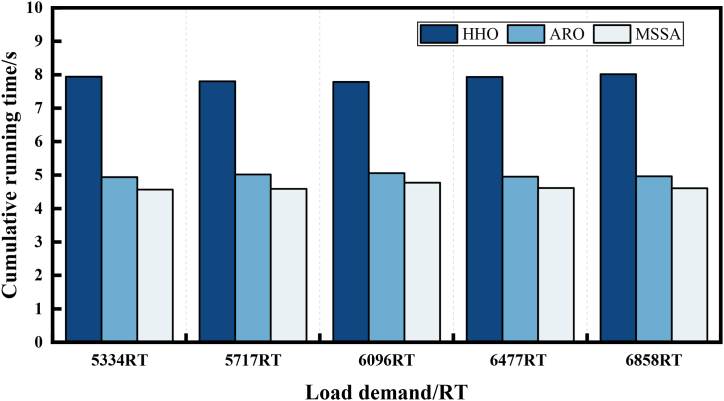
Cumulative running time of 3 algorithms of Case 1.

**Fig. 14:**
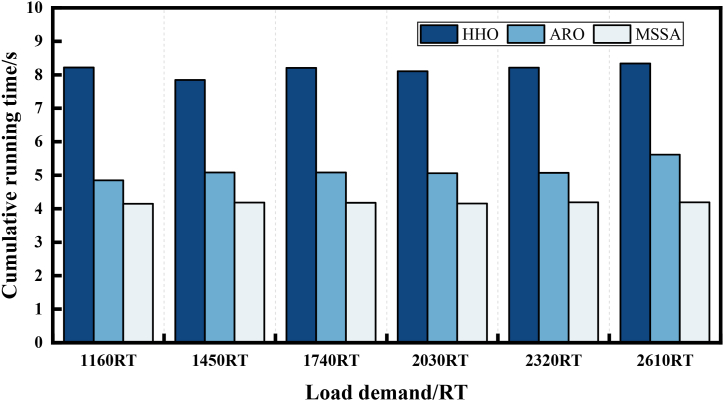
Cumulative running time of 3 algorithms of Case 2.

In optimization problems, an excellent algorithm should exhibit low computational complexity. We employed the cumulative running time of 30 operations to assess the computational complexity of the three algorithms. Fig. 13 illustrates the cumulative running time of the three algorithms across all load demand tests in Case 1. HHO solutions take approximately 7.78s–8.02s, ARO takes 4.94s–5.06s, and MSSA only demands 4.57s–4.77s over the five load demand conditions. The shortest solution time of MSSA indicates the lowest computational complexity. For Case 2, the cumulative running time of the MSSA is still the smallest among the three algorithms. Moreover, Fig. 14 reveals that compared to the HHO algorithm, MSSA algorithm solution time is reduced by approximately 50 %. The two cases comprehensively demonstrate that MSSA is significantly fast and can respond to OCL problems in a shorter time compared to HHO and ARO.

### 5.5 Robustness analysis

**Fig. 15:**
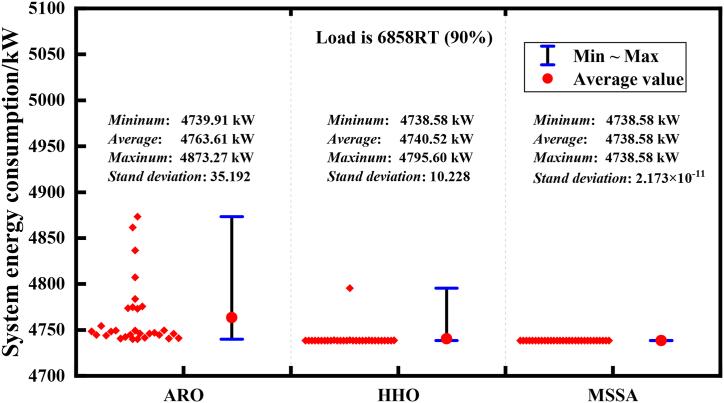
Robustness analysis of 3 algorithms of Case 1 (load is 6858RT).

**Fig. 16:**
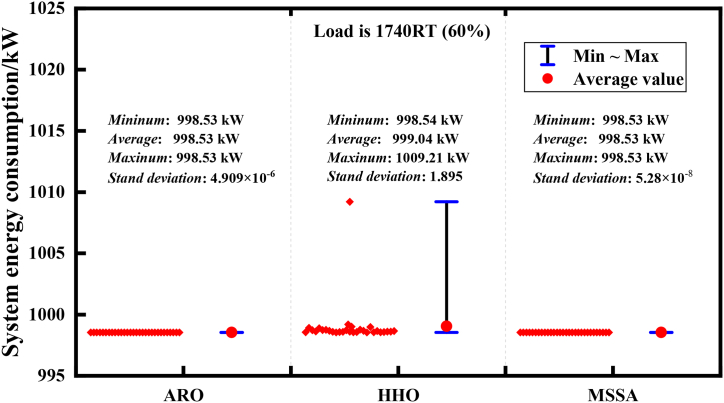
Robustness analysis of 3 algorithms of Case 2 (load is 1740RT).

In addition, to conduct a comprehensive analysis of MSSA, Fig. 15, Fig. 16 present the range of solutions for the three algorithms in 30 operations.

In Case 1, Fig. 15 indicates that when the end-energy demand is 6858RT under high load conditions, after 30 independent operations, ARO algorithm solutions exhibit a relatively dispersed range, varying from 4873.27 kW to 4739.91 kW, and its standard deviation reaches 35.192, which indicates unstable performance. The solution range of the HHO algorithm, ranging from 4738.58 kW to 4795.60 kW, is relatively stable compared to ARO. The MSSA algorithm exhibits the smallest difference range between the maximum value and the minimum value, and its standard deviation is also close to 0, indicating excellent robustness. As shown in Fig. 16, when the load demand is 1740RT in Case 2, both MSSA and ARO algorithms exhibit standard deviations close to 0, with MSSA slightly outperforming ARO. However, the solution range of the HHO algorithm, which ranges from 998.54 kW to 1009.21 kW, demonstrates less robustness than MSSA and ARO. The tests of the two conditions have strongly proved that MSSA has excellent robustness. However, by analyzing the reasons, we believe that because scroungers occupy a large proportion in the sparrow population, its position update is affected by producers. The introduction of Cauchy variation strategy disturbs scroungers' position update and reduces the possibility of MSSA obtaining the local optimal value. When MSSA's global search ability is improved, every solution can get a very competitive optimal solution, so that the robustness of MSSA has been greatly enhanced.

## 6 Conclusions and future work

To tackle the OCL problem effectively, this paper proposes a multi-strategy improved sparrow search algorithm. Through testing two typical cases, we draw the following conclusions.

(1) After testing the benchmark function and comparing the results with PSO, HHO, ARO and SSA algorithms, we believe that the proposed MSSA has significant optimization ability.
(2) In the test of solving OCL problem, two classical cases show that the proposed MSSA can provide a competitive energy saving scheme. Compared to GA, PSO and SSA algorithms, for Case 1, when the load demand is 1160RT, MSSA can provide a maximum energy-saving potential of 205.23 kW, which has a significant energy-saving impact. Furthermore, the test results of both cases demonstrate that the lower the end-building energy demand, the greater the energy-saving potential of MSSA.
(3) For OCL problems, more test results illustrate that MSSA has significant advantages in convergence speed, computational complexity and robustness, which preliminarily proves the effectiveness of MSSA in solving such problems.

In summary, the proposed MSSA can effectively to optimal chillers loading, and the use of metaheuristic optimization algorithms provides a new approach to investigate efficient building energy-saving methods. However, we also found that MSSA has a lot of room for improvement in terms of robustness. In our future work, we will utilize new strategy to enhance MSSA to solve OCL problems, and try to apply MSSA to other fields of engineering optimization problems. In addition, we will also be committed to the research of multi-objective MSSA algorithm, and establish a multi-objective energy optimization model based on economy, energy efficiency, carbon emission, etc., to provide new ideas and methods for other energy optimization problems.

## Data availability statement

Data will be made available on request.

## CRediT authorship contribution statement

**Xiaodan Shao:** Data curation, Formal analysis, Investigation, Methodology, Software, Validation, Visualization, Writing – original draft. **Jiabang Yu:** Formal analysis, Investigation, Validation, Writing – original draft. **Ze Li:** Formal analysis, Investigation, Software, Writing – original draft. **Xiaohu Yang:** Conceptualization, Funding acquisition, Methodology, Project administration, Supervision, Writing – review & editing. **Bengt Sundén:** Conceptualization, Supervision, Writing – review & editing.

## Declaration of competing interest

The authors declare that they have no known competing financial interests or personal relationships that could have appeared to influence the work reported in this paper.
